# Deregulated lncRNAs in B Cells from Patients with Active Tuberculosis

**DOI:** 10.1371/journal.pone.0170712

**Published:** 2017-01-26

**Authors:** Yurong Fu, Xianqin Xu, Junfang Xue, Wenping Duan, Zhengjun Yi

**Affiliations:** 1 Department of Medical Microbiology of Clinical Medicine College, Weifang Medical University, Shandong, Weifang, China; 2 Department of Laboratory Medicine, Key Laboratory of Clinical Laboratory Diagnostics in Universities of Shandong, Weifang Medical University, Shandong, Weifang, China; 3 Department of Clinical Skill Laboratory of Clinical Medicine College, Weifang Medical University, Shandong, Weifang, China; 4 Department of Surgery of Gaomi City people's Hospital, Shandong, Weifang, China; 5 Department of Nursing of Affiliated Hospital of Weifang Medical University, Shandong, Weifang, China; Kunming University of Science and Technology, CHINA

## Abstract

Role of lncRNAs in human adaptive immune response to TB infection is largely unexplored. To address this issue, here we characterized lncRNA expression profile in primary human B cell response to TB infection using microarray assay. Several lncRNAs and mRNAs were chosen for RT-qPCR validation. Bioinformatics prediction was applied to delineate function of the deregulated mRNAs. We found that 844 lncRNAs and 597 mRNAs were differentially expressed between B cell samples from individuals with or without TB. KEGG pathway analysis for the deregulated mRNAs indicated a number of pathways, such as TB, TLR signaling pathway and antigen processing and presentation. Moreover, corresponding to the dysregulation of many lncRNAs, we also found that their adjacent protein-coding genes were also deregulated. Functional annotation for the corresponding mRNAs showed that these lncRNAs were mainly associated with TLR signaling, TGF-β signaling. Interestingly, SOCS3, which is a critical negative regulator of cytokine response to TB infection and its nearby lncRNA XLOC_012582, were highly expressed in active TB B cells. Subsequent RT-qPCR results confirmed the changes. Whether upregulated XLOC_012582 causes SOCS3 overexpression and is eventually involved in the context of exacerbations of active TB represents an interesting issue that deserves to be further explored. Taken together, for the first time, we identified a set of deregulated lncRNAs in active TB B cells and their functions were predicted. Such findings provided novel insight into the pathogenesis of TB and further studies should focus on the function and pathogenic mechanisms of the lncRNAs involved in active TB.

## Introduction

Tuberculosis (TB), caused by *Mycobacterium tuberculosis* (Mtb), remains a major challenge to human public health [1]. According to the World Health Organization, there are reported to be more than 30% of population infected with Mtb. Host immune response against Mtb is complex and multifaceted. The underlying mechanisms of TB pathogenesis are still poorly understood, especially the mechanisms explaining how the immune system dysfunction in TB development [2]. Accordingly, the elucidation of molecular mechanisms in TB has been the subject of extensive research over past decades. Better understanding of the interplay between Mtb and host immune response is critical for TB control and prevention [3].

Effective cell-mediated immune response plays an essential role in the host defense against intracellular pathogen Mtb[4,5]. Immunity to TB is still understood to be driven and maintained by T-cell derived immune response [6]. However, B cells, as one of the major adaptive immune cells, are thought to play a limited role [7]. Recent evidence shows that B cells can regulate immune response to Mtb via the modulation of cytokine production and macrophage activation [8]. Moreover, one most recent study indicates that human B cells can phagocytose Mtb, which can in turn regulate the immune activation of B cells [9]. The data suggest that B cells, as effectors in both the innate and adaptive immune response, can modulate host defense against Mtb infection and play a significant role in determining the clinical outcome of TB infection [10]. Despite this, the contributory role of B cells in the protection against Mtb infection is less defined than the role of T cells.

Increasing evidence reveals that long non-coding RNAs (lncRNAs) have key functions in regulating diverse biological processes, such as transcription activation and inhibition, mRNA translation, organellar biogenesis as well as cell development [11]. LncRNAs are responsible for at least 80% of all genome transcripts and have been shown to be involved in various pathophysiologic processes and human diseases. Accumulated evidence also indicates that lncRNA plays an important role in host immune responses against invading pathogens and its misregulation has been shown in different types of infectious diseases [12]. For instance, lncRNA NRON can modulate HIV-1 replication in a NFAT-dependent manner [13]. We previously found that many lncRNAs were differentially expressed in CD4^+^ T cells from patients with active TB [14]. Recently, one study shows that downregulated lncRNA MEG3 eliminates mycobacteria in macrophages via autophagy [15] and another study indicates that lncRNA BC050410 inhibits CD8^+^ T-cell immune response in TB infection [16]. A more recent study reveals significantly altered lncRNA expression profiles in plasma from patients with TB disease [17] and another study shows that two lncRNAs, MIR3945HG V1 and MIR3945HG V2, are identified as novel candidate diagnostic markers for TB [18]. The data suggest that similar to its important role in other infectious diseases, lncRNA is demonstrated to be closely associated with Mtb infection. What remains to be seen is whether there exists an altered lncRNA profile in the active TB B cells.

Hence, the present study aimed to explore the expression profiles of lncRNA and mRNA in the B cells obtained from subjects with or without active TB, and deregulated lncRNAs and mRNAs were also evaluated in independent patient and control samples.

## Materials and Methods

### Human subjects

Patients with active pulmonary TB (male/female = 13:18) with a mean age of 42.1±15.5 yr (ATB group) were recruited from Weifang No. 2 People’s Hospital of Shandong Province, China. Diagnosis of active pulmonary TB was based on both sputum smear and culture positive or at least one sputum culture positive, as well as typical pulmonary TB clinical symptoms. Healthy subjects (male/female = 15:20) with a mean age of 40.3±13.9 yr (control group) were enrolled from the staff of Weifang Medical University, China, were free of clinical symptoms of any infectious disease, and had no close contact of a TB patient. All involved subjects had no history of TB, diabetes, tumor or other infectious disease. Three samples randomly selected from each group (male/female = 1:2) were used in the microarray assay and all of the samples were used for further RT-qPCR confirmation.

The study was conducted in accordance with the Declaration of Helsinki. Written informed consent was obtained from each participant prior to enrollment. The study was approved by the Research Ethics Committee of Weifang Medical University, China (Consent NO.: 2013–054).

### Isolation of B cells

For preparation of B cells, fasting venous blood was drawn from each subject into a coded EDTA-anticoagulant tube. Peripheral blood mononuclear cells (PBMCs) were collected from venous blood using density gradient centrifugation. B cells were then isolated from obtained PBMCs by negative selection using B Cell Isolation Kit (R&D Systems, Minneapolis, MN, USA) according to the manufacturer’s instructions. Briefly, B cells were negatively selected by depletion of unwanted cells. Flow cytometric analysis clearly showed that the purity of isolated B cells was over 90% (S1 Fig). Qualified samples were immediately stored in liquid nitrogen until further use.

### RNA extraction and RNA quality control

For RNA preparation, total RNA was isolated from purified B cells using TRIzol^®^ Reagent (Invitrogen, Carlsbad, CA, USA) and further purified with RNeasy mini kit (Qiagen, Hilden, Germany). Quantification and quality check were performed with Nanodrop ND-1000 (NanoDrop Technologies,Wilmington, USA) and Agilent 2100 Bioanalyzer (Agilent Technologies Europe, Waldbroon, Germany), respectively. RNA integrity was assessed by standard denaturing gel electrophoresis. Only RNA sample with good quality was used for further downstream processing.

### Microarray analysis of lncRNAs and mRNAs

For gene chip hybridization, RNA sample labeling and array hybridization were performed using Quick Amp Labeling Kit, One-Color (Agilent, Santa Clara, CA, USA) according to the manufacturer’s instructions. Briefly, mRNA sample was purified from total RNA after removal of rRNA with mRNA-ONLY^™^ Eukaryotic mRNA Isolation Kit (Epicentre, Madison, WI, USA). Each sample was then transcribed to double-stranded cDNA, synthesized into cRNA and subsequently labeled with Cyanine-3-CTP. The labeled samples were then purified using RNAeasy Mini Kit (Qiagen, Hilden, Germany). The yield and specific activity of labeled cRNAs were then measured by NanoDrop ND-1000. Only if the yield is over 1.65 μg and the specific activity is more than 9.0 pmol Cy3 per μg cRNA, can labelled cRNAs proceed to next hybridization step. After passing quality test, 1 μg of each labeled cRNAs in hybridization solution was used for hybridization on Human LncRNA Microarray v3.0 (Arraystar, Rockville, MD, USA), which contains probes of 30,586 human lncRNAs and 26,109 human protein-coding transcripts. All the lncRNAs were obtained from authoritative databases, GENCODE, UCSC Knowngene, RefSeq, UCR and many related literatures. Positive control probes for 28 housekeeping genes (NM_003753, NM_005022, NM_002046, NM_002539, NM_001861, NM_001101, NM_001614, NM_000841, NM_006098, NM_022551, NM_001536, NM_000291, NM_002107, NM_021009, EIF3D, PFN1, GAPDH, ODC1, COX4I1, ACTB, ACTG1, GRM4, GNB2L1, RPS18, PRMT1, PGK1, H3F3A and UBC) and negative control probes were used for hybridization quality control. After hybridization, each microarray slide was washed and immediately scanned using an Agilent Microarray Scanner (Agilent G2505C, Santa Clara, CA, USA).

### Data analysis

Agilent Feature Extraction software (version 11.0.1.1) was used to analyze acquired array images. Quantile normalization and subsequent data processing were performed using GeneSpring GX v12.1 software package (Agilent Technologies). After quantile normalization of the raw data, lncRNAs and mRNAs that at least 3 out of 6 samples have flags in Present or Marginal (all targets value) were chosen for further data analysis. Differentially expressed lncRNAs and mRNAs with statistical significance between active TB group and healthy controls were identified through *P-*value corrected using the false discovery rate method. The threshold set for differentially expressed genes was fold-change >2.0 (*P*<0.05). A positive fold-change value indicates upregulation and a negative fold-change value indicates downregulation. Moreover, hierarchical clustering was performed to display the distinguishable lncRNA and mRNA expression patterns among the samples.

In addition, subgroups of the deregulated lncRNAs, including large intervening noncoding RNAs (lincRNAs), lncRNAs with enhancer-like function, antisense lncRNAs as well as their paired differentially expressed mRNAs, were also identified,

### GO and KEGG pathway analysis

Gene Ontology (GO) project provides a controlled vocabulary to describe gene and gene product attributes (http://www.geneontology.org) [19]. For GO enrichment analysis of the differentially expressed mRNAs, GO categories are considered as significantly enriched only if *P*-value < 0.05. Kyoto Encyclopedia of Genes and Genomes (KEGG) enrichment analysis is used to analyze involved biological pathways of the deregulated mRNAs. The *P*-value denotes the significance of the pathway correlated to the conditions. The lower the *P*-value, the more significant considered the pathway.

### RT-qPCR analysis

The expression pattern of selected lncRNAs and mRNAs in all samples was analyzed with a SYBRGreen PCR kit (TaKaRa, Dalian, China). Primers were designed and synthesized by Generay Biotech (Shanghai, China). The thermal cycling conditions for PCR reaction were as follows: initial denaturation at 95°C for 10 min, followed by 40 cycles at 95°C for 10 s, 60°C for 60 s and 72°C for 15 s. All RT-qPCR experiments included no-template controls. Each sample was detected in triplicate. Glyceraldehyde 3-phosphate dehydrogenase (GAPDH) mRNA was used as an internal control and expression level of each RNA was normalized to that of GAPDH. Fold change of RNA expression in ATB versus control group was calculated using 2^-*△△*Ct^ method and a *P*-value of less than 0.05 was considered statistically significant.

### Statistical analysis

All data were presented as mean ± standard deviation (SD) or proportions where appropriate. Student’s *t*-test or chi-square test was used for statistical analysis where appropriate. *P* < 0.05 was considered statistically significant.

## Results

### Profile of microarray data

Expression profiling studies were performed on RNA from three independent B samples from each group. The microarray expression data discussed in the article have been deposited into National Center for Biotechnology Information (NCBI) Gene Expression Omnibus (GEO) and are accessible through (GEO) Series accession number GSE89552. In total, 844 lncRNAs were identified with differential expression between samples from subjects with or without active TB (fold change > 2.0, *P*<0.05), of which 345 lncRNAs were increased and 499 lncRNAs were decreased in samples from active TB patients, respectively. Among them, ENST00000505706 was the top increased lncRNA and ENST00000562027 was the most decreased ones.

**Fig 1:**
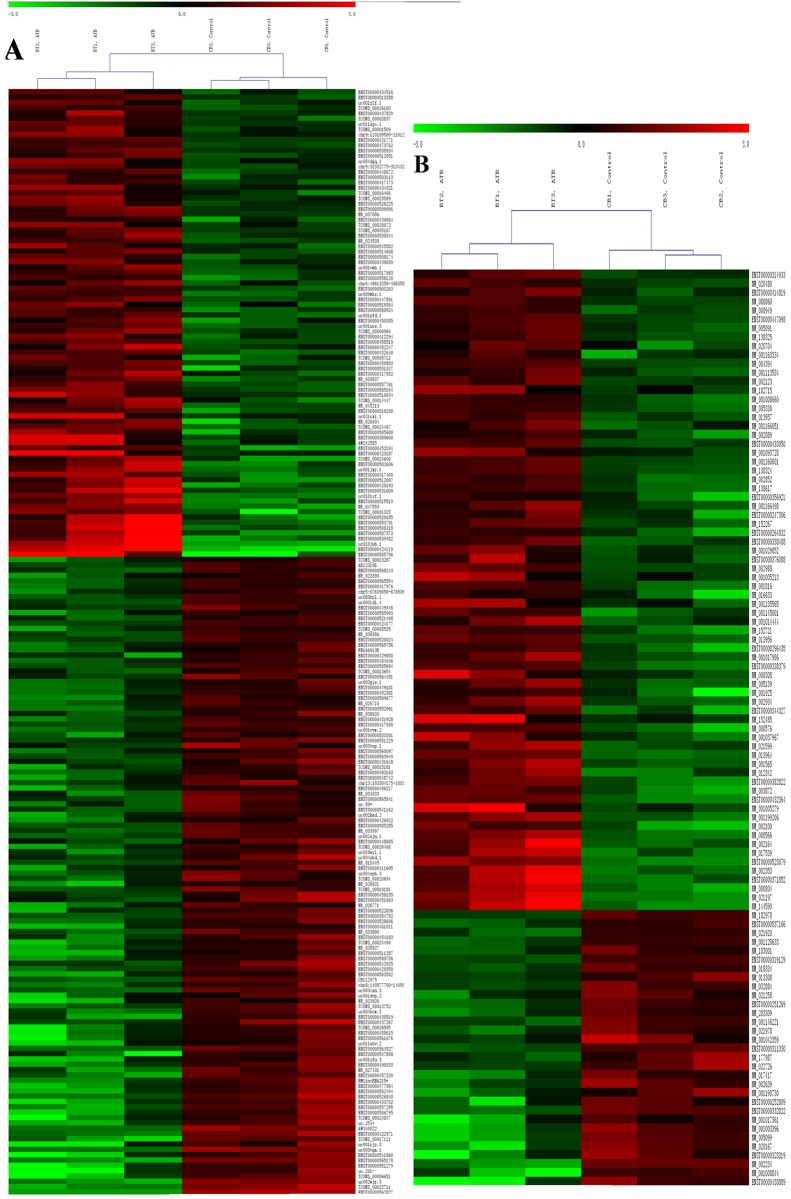
Differentially expressed lncRNAs (A) and mRNAs (B) between ATB group and control group. Red indicated high relative expression and green indicated low relative expression. LncRNA or mRNA with expression fold change > 3 and with FDR adjusted *P* value < 0.05 was considered statistically significant. ATB group: BT1, BT2, BT3; Control group: CB1, CB2, CB3.

Further data analysis showed that 361 upregulated and 236 downregulated mRNAs were also identified in ATB group compared with healthy controls, of which 118 lncRNAs and 72 mRNAs exhibited fold changes > 3.0 (Fig 1). Among them, ANKRD22 was the top upregulated mRNA and HOXC4 was the most downregulated ones. Numerous studies indicate that a variety of cellular events are disturbed in the progression of TB, ranging from matrix synthesis to cytokine expression[20]. Underlying these alterations there is the dysregulated gene expression of particular molecules. Our data suggested that molecular events in peripheral blood B cells, such as lncRNAs and mRNAs, were altered during active TB infection.

### Expression signatures of the deregulated lncRNAs

**Fig 2:**
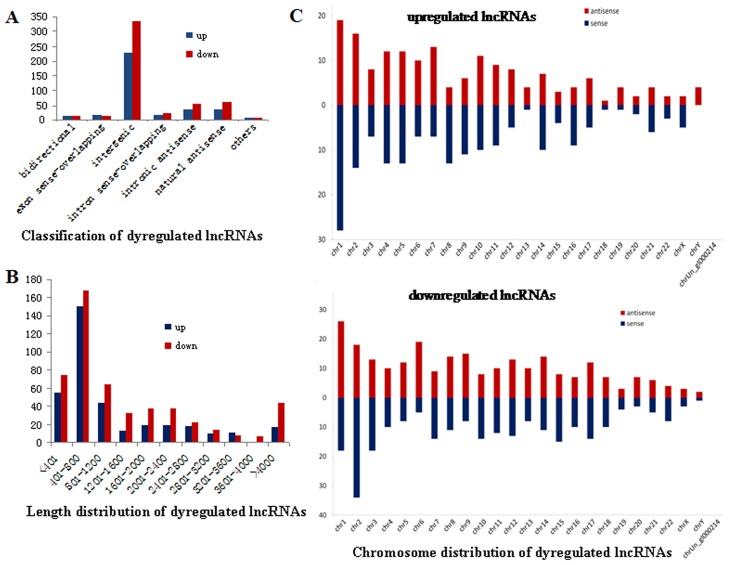
Expression signatures of deregulated lncRNAs in ATB group versus control group. (A) Classification of lncRNAs. They were mainly intergenic. (B) Length distribution of lncRNAs. They were mainly between 401 and 800 nt in length. (C) Chromosome distribution of lncRNAs. Chromosome 1 and 2 were the most frequent ones and numbers of the deregulated lncRNAs on the two chromosomes accounted for approximately 11% and 9% of total deregulated ones, which were higher than the expected numbers based on chromosome total lncRNA numbers, respectively.

As lncRNA expression is cell specific [21], to further study the lncRNA expression pattern in B cells associated with active TB, general signatures of the deregulated lncRNAs were investigated, including lncRNA classification, length and chromosome distribution. According to the positional relationship between lncRNA and its adjacent protein-coding genes in the same chromosome, lncRNAs can be roughly classified as bidirectional, intron sense-overlapping, exon sense-overlapping, intergenic, intronic antisense and natural antisense. The majority of deregulated lncRNAs in the study were related to intergenic (~63%) (Fig 2A) and distributed 401–800 nt (~38%) in length (Fig 2B). Chromosome distribution of the deregulated lncRNAs showed that chromosome 1 and 2 were the most frequent ones and numbers on these two chromosomes accounted for approximately 11% and 9% of total deregulated lncRNAs, which were higher than the expected numbers based on chromosome total lncRNA numbers, respectively (Fig 2C). The variation of lncRNAs expression in human B cells indicated that these deregulated lncRNAs may be involved in the onset and development of active TB.

### GO analysis and pathway analysis of deregulated mRNAs

GO and KEGG pathway analysis were used to analyze the potential roles that the differentially expressed mRNAs played in GO biological process and pathways. GO analysis of the deregulated mRNAs in the study identified numerous biological processes with significantly altered expression of gene products involved. Many of these processes which are upregulated in active TB B cells are mainly involved in single organism process, immune system process, immune response, response to bacterium and molecule of bacterial origin. In contrast, other processes which are downregulated in active TB B cells are related to immune activation, such as T cell activation, T cell differentiation, positive regulation of lymphocyte mediated immunity and cytotoxicity, positive regulation of leukocyte mediated immunity and natural killer cell mediated cytotoxicity (Table 1). KEGG pathway analysis showed that 23 pathways corresponded to increased mRNAs and 8 pathways corresponded to decreased mRNAs, respectively. The top 10 pathways associated with overexpressed mRNAs were primarily enriched in TB, phagosome, toll like receptor signaling pathway and TNF signaling pathway. However, enriched pathways of underexpressed mRNAs were mainly related to antigen processing and presentation, T cell receptor signaling pathway as well as natural killer cell mediated cytotoxicity (Table 1). The data demonstrated that B cell responses to active TB infection were differentially modulated and suppressed.

**Table 1:** GO and pathway analysis of upregulated mRNAs and downregulated mRNAs.

GO Term	*P* value	KEGG Pathway	*P* value
response to bacterium	1.72E-12	osteoclast differentiation	2.52E-06
response to wounding	3.53E-11	Leishmaniasis	1.84E-05
cell motility	1.71E-10	*Staphylococcus aureus* infection	0.00015
localization of cell	1.71E-10	rheumatoid arthritis	0.00260
locomotion	2.88E-10	tuberculosis	0.00374
immune system process	3.09E-10	phagosome	0.00457
cell migration	3.91E-10	chagas disease (American trypanosomiasis)	0.00548
immune response	4.07E-10	platelet activation	0.00548
single-organism process	8.75E-09	toll-like receptor signaling pathway	0.00607
response to molecule of bacterial origin	1.43E-08	TNF signaling pathway	0.00741
*T cell differentiation*	*4*.*83E-05*	*Inflammatory bowel disease*	*0*.*00102*
*alpha-beta T cell differentiation*	*0*.*00018*	*antigen processing and presentation*	*0*.*00214*
*regulation of cell fate commitment*	*0*.*00033*	*natural killer cell mediated cytotoxicity*	*0*.*00447*
*positive regulation of lymphocyte mediated immunity*	*0*.*00040*	*measles*	*0*.*00447*
*lymphocyte activation*	*0*.*00044*	*hematopoietic cell lineage*	*0*.*01812*
*positive regulation of leukocyte mediated immunity*	*0*.*00047*	*acute myeloid leukemia*	*0*.*02815*
*T cell activation*	*0*.*00053*	*T cell receptor signaling pathway*	*0*.*03233*
*organ development*	*0*.*00062*	*chagas disease (American trypanosomiasis)*	*0*.*03233*
*positive regulation of leukocyte mediated cytotoxicity*	*0*.*00068*		
*alpha-beta T cell activation*	*0*.*00074*		

Italic: enriched GO Term and KEGG pathway of downregulated mRNAs. Each *P*-value denoted the significance of the GO Term or KEGG pathway. The lower the *P*-value, the more significant GO Term or the pathway was.

### Confirmation of the microarray results by RT-qPCR

**Fig 3:**
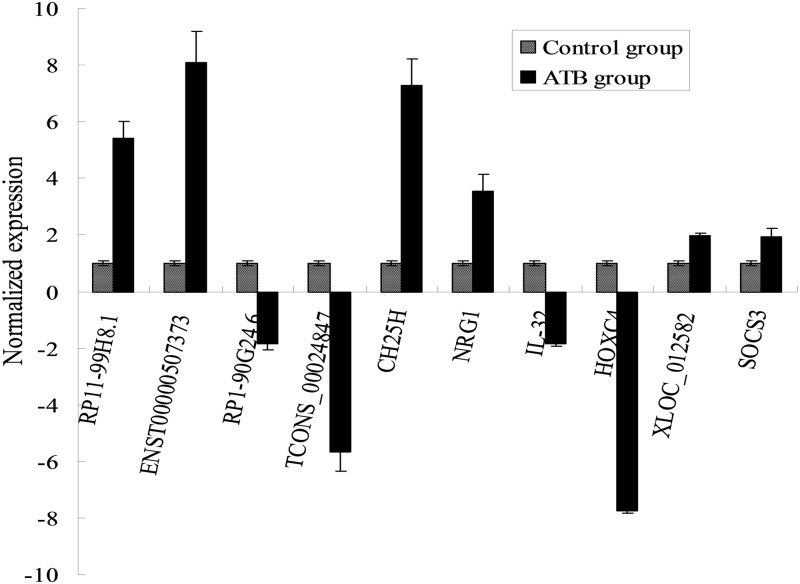
RT-qPCR validation results of the randomly selected lncRNAs and mRNAs between ATB group and control group. Four lncRNAs RP11-99H8.1, ENST00000507373, RP1-90G24.6, TCONS_00024847 and four mRNAs CH25H, NRG1, IL-32, HOXC4 were chosen for the validation of the microarray data usingRT- qPCR. The height of the columns in the figure represents the lncRNA expression level relative to GAPDH; the bars represent SD. *P <0.05 versus control group.

Four deregulated lncRNAs RP11-99H8.1, ENST00000507373, RP1-90G24.6, TCONS_00024847 as well as four mRNAs CH25H, NRG1, IL-32 and HOXC4 were randomly selected to confirm the microarray data in all samples from ATB group and control group (Fig 3). The results showed that RP11-99H8.1, ENST00000507373,CH25H and NRG1 were increased, while RP1-90G24.6, TCONS_00024847, IL-32 and HOXC4 were decreased in ATB versus control samples (*P*<0.05). The RT-qPCR results matched well with the microarray data, which demonstrated high credibility for the microarray analysis.

### Subgroup analysis of the differentially expressed lncRNAs and their adjacent mRNA pairs

In the study, the differentially expressed lncRNAs and their neighboured protein-coding genes were focused. It was noteworthy that 9 antisense lncRNAs with their adjacent mRNA pairs were identified as coregulated transcripts, of which 8 pairs showed similar expression direction (upregulated or downregulated) (Table 2). Moreover, there were 11 pairs of aberrantly expressed enhancer-like lncRNAs and their adjacent mRNAs (distance < 300 kb) (fold-change > 2, *P* < 0.05), of which 6 pairs were differentially expressed in similar direction (increased or decreased) (Table 3). In addition, profiling data based on Rinn’s lincRNAs indicated that there were 21 aberrantly expressed lincRNAs with their deregulated associated protein-coding genes (fold-change > 2, *P* < 0.05), of which 16 pairs were deregulated in similar direction (overexpressed or underexpressed) (Table 4). Of these, expression of XLOC_012582 lncRNA and its paired SOCS3 mRNA was further confirmed by RT-qPCR in the study (Fig 3). Detailed relationship on genomic location between these lncRNA and the adjacent protein-coding genes was shown in S1 Table.

**Table 2:** Deregulated antisense lncRNAs and their associated coding genes.

GeneSymbol	Fold change—lncRNAs	source	RNA length	Genome Relationship	NearbyGene Symbol	Fold change—mRNAs
CHRM3-AS2	-2.61	GENCODE	950	intronic antisense	CHRM3	-2.09
RP11-153M7.5	+2.19	GENCODE	521	intronic antisense	RNF175	-2.106
HLA-DQB1-AS1	+2.87	GENCODE	552	natural antisense	HLA-DQB1	+3.27
RP11-196G18.3	+2.50	GENCODE	3543	natural antisense	FCGR1A	+6.76
RP11-439A17.9	+2.89	GENCODE	3543	natural antisense	FCGR1B	+2.94
RP11-439A17.9	+2.89	GENCODE	3543	natural antisense	FCGR1B	+4.49
RP11-597D13.9	+2.32	GENCODE	692	natural antisense	FAM198B	+2.38
RP11-597D13.9	+2.32	GENCODE	692	natural antisense	FAM198B	+2.98
RP11-244F12.2	+2.33	GENCODE	691	natural antisense	TPM1	+2.05

For the differentially expressed lncRNAs and mRNAs, all *P*<0.05 in ATB group versus control group.

**Table 3:** Deregulated enhancer-like lncRNAs and nearby coding genes (distance<300 kb).

Gene Symbol	Fold change—lncRNAs	source	RNA length	Genome Relationship	NearbyGeneSymbol	Fold change—mRNAs
RP11-99H8.1	+6.34	GENCODE	432	upstream	PTGER3	+3.07
RP11-478H13.3	-3.61	GENCODE	406	upstream	BAMBI	+5.69
RP11-109A6.4	-2.22	GENCODE	2705	downstream	EBF3	+2.18
KIAA0664L3	-2.79	GENCODE	723	upstream	AHSP	+4.00
RP1-122P22.2	+2.24	GENCODE	287	downstream	C20orf26	+2.09
AC109826.2	-2.67	GENCODE	548	downstream	MGAT4A	-2.05
AC109826.2	-2.67	GENCODE	548	downstream	INPP4A	-2.02
RP1-90G24.6	-2.40	GENCODE	590	downstream	RFPL3	-2.82
RP1-90G24.6	-2.40	GENCODE	590	downstream	SYN3	-2.01
KIAA0664L3	-2.90	GENCODE	1907	upstream	AHSP	+4.00
LINC00324	-2.17	RefSeq	2115	downstream	ALOX15B	+2.14

For the differentially lncRNAs and mRNAs, all *P*<0.05 in ATB group versus control group.

**Table 4:** Deregulated lincRNAs and associated coding gene pairs (distance<300 kb).

Gene Symbol	Fold change—lncRNAs	RNA length	Genome Relationship	NearbyGene Symbol	Fold change—mRNAs
XLOC_000218	+3.94	730	upstream	TACSTD2	+9.11
XLOC_000166	+2.09	883	downstream	PTCH2	+3.14
XLOC_000647	-2.04	1073	downstream	NLRP3	+2.75
XLOC_002993	+4.05	204	downstream	MUC4	+2.09
XLOC_002993	+4.05	204	downstream	MUC4	+2.14
XLOC_002813	+2.50	1126	downstream	IFT122	-2.12
XLOC_004199	-2.25	211	downstream	CLDN22	-7.19
XLOC_006188	+2.45	233	upstream	AZGP1	+2.03
XLOC_007896	-2.26	371	upstream	FAM69B	+2.02
XLOC_007896	-2.42	703	upstream	FAM69B	+2.02
XLOC_008811	-2.81	4872	downstream	ZNF488	-2.12
XLOC_008811	-2.81	4872	upstream	PTPN20B	-3.75
XLOC_009016	-3.83	1239	downstream	SCT	-3.08
XLOC_009281	-2.00	374	downstream	ZBTB16	-2.22
XLOC_009734	+2.25	276	downstream	OR8S1	+2.40
XLOC_010052	+2.62	2707	upstream	LRRK2	+2.24
XLOC_010898	-2.40	486	downstream	KCNK10	+2.21
XLOC_011893	+6.39	219	downstream	DNAH3	+7.92
XLOC_011893	+6.39	219	upstream	CRYM	+4.28
XLOC_012582	+2.06	1636	downstream	SOCS3	+2.13
XLOC_012626	-2.10	1743	downstream	GNAL	-3.05

For the differentially lncRNAs and mRNAs, all *P*<0.05 in ATB group versus control group.

It has been shown that transcription of lncRNAs can affect expression of their nearby coding genes at the level of chromatin modification, transcription and post-transcriptional processing, and their dysregulation was involved in many diseases [22–24]. In the study, not all pairs of IncRNAs and mRNAs are changed in the same direction. LncRNAs have the ability to regulate transcription by affecting gene promoters through interacting with initiation complex [25]. Moreover, some lncRNAs, like antisense lncRNAs, can influence mRNAs expression through splicing, editing and translation in the post-transcriptional processing [26]. Generally, an equidirectional transcriptive target gene is for promoting expression in the promoter region, otherwise it is for suppression. In some conditions, a reversed direction is possible to promote expression in the 3′-UTR region [27]. Our data suggested that these deregulated lncRNAs may positively or negatively regulate their adjacent mRNAs expression and by which, they may affect B cell function and so contribute to the pathogenesis of active TB. The differential expression of lncRNA and its relationship with the protein-coding genes are of great significance in active TB. Further studies were needed to confirm these lncRNAs functions with knockdown and over-expression experiments.

## Discussion

The molecular determinants of B cell immune response to TB are largely unknown. Only a handful of studies to-date have examined lncRNA expression in TB disease. In the current study, we demonstrated that there was a significantly altered lncRNA and mRNA expression profile in the active TB B cells *in vivo*. We identified 844 lncRNAs and 597 mRNAs with differential expression between TB and non-TB B cells and we confirmed a selection of these differentially expressed transcripts by RT-qPCR. GO and KEGG pathway analysis of the deregulated mRNAs showed that biological function and signaling pathway were altered in B cells and positive regulation of B cell response against TB infection was also changed.

The aberrantly expressed lncRNAs observed in the study may provide clues to the dysfunction of B cells and so to the pathophysiological properties of active TB. However, their corresponding functions remain poorly understood. It has been shown that the transcription of lncRNAs can affect the expression of their nearby coding gene [28]. In the study, we analyzed lncRNAs and their associated protein-coding genes, which could help to predict and reveal the function and mechanism of lncRNAs in active TB. Corresponding to the dysregulation of many lncRNAs, we also found that their adjacent protein-coding genes were also deregulated. The following discussion was mainly focused on these paired lncRNA and mRNAs. Functions of most associated aberrantly expressed mRNAs in active TB were also little known, hence, we discussed these lncRNAs based on the function of their associated mRNAs reported in other studies.

Our results showed that expression of 7 antisense lncRNAs was correlated with that of corresponding nearby mRNAs. Although the corresponding functions of these mRNAs in active TB remain largely unknown, based on other data, we can find that they are mainly involved in cell proliferation (CHRM3), protein ubiquitination (RNF175), genetic susceptibility (HLA-DQB1), antibody function (FCGR1A, FCGR1B), T-cell homeostasis (FAM198B) and promoter activity (TPM1) [29,30]. Moreover, nine enhancer-like lncRNAs are associated with 11 significantly differentially expressed mRNAs in the study. These mRNAs are related to TLR signaling (PTGER3), TGF-β signaling (BAMBI), cell division, growth or death (EBF3, INPP4A, RFPL3, SYN3), oxidative stress (AHSP), cell cycle (MGAT4A), lipid metabolism and inflammation (ALOX15B) [31,32]. However, the mechanisms by which they are connected to the designated lncRNAs and so to the pathogenesis of active TB remain unknown. The lncRNAs and related gene pathways detected in our study suggest the complicated molecular mechanism of active TB.

In addition, 17 differentially expressed lincRNAs and associated coding gene pairs were identified in the study. These associated protein-coding genes are linked to cell apoptosis (TACSTD2), notch and hedgehog signaling (PTCH2), cell survival or division (MUC4, IFT122), inflammasome response (NLRP3), tumor suppressor (CLDN22), TGF-β signaling (AZGP1), phosphotransferase activity (FAM69B), WNT/β-catenin signaling (ZNF488), autophagy (ZBTB16, LRRK2) and cell differentiation (KCNK10). SOCS3, a critical negative regulator of STAT3-dependent cytokine response, is one major controller of the outcome of TB infection[33]. Many reports have shown that TB progression is associated with increased SOCS3 in T cells [34], macrophages[35] and bronchoalveolar lavage [36]. We demonstrated here for the first time that SOCS3 was also upregulated in active TB B cells. Interestingly, we found that lincRNA XLOC_012582, located on the upstream of SOCS3, was also significantly increased in the same samples. Expression of SOCS3 mRNA and XLOC_012582 lncRNA was further confirmed by RT-qPCR in the study. This may imply a partial increase of regulation of SOCS3 expression, and although very little is known about the functions of this lncRNA, this transcript may hold relevance in the context of exacerbations of active TB, which represents an interesting issue that deserved to be further explored.

In summary, our result for the first time showed that many lncRNAs were differentially expressed in active TB B cells, and their functions could be predicted based on their positional relation with protein-coding genes. Although available datasets in the study were limited and these lncRNA signatures need further identification and validation, worth noting is the fact that the aberrant lncRNAs may play important role in misregulation of B cell response against TB infection, which in turn may affect TB development. These findings shed a novel light on the pathogenesis of TB and provide a basis for the diagnosis and therapy of TB.

## Supporting Information

S1 Fig Flow cytometric analysis of the purity of isolated B cells. PBMCs before (A) and after (B) isolation of B cells.
(TIF)

S1 Table Positional relationship between lncRNA and the adjacent protein-coding genes. (XLS)

## References

[1] Zheng L, Leung E, Lee N, Lui G, To KF, Chan RC, et al Differential microRNA expression in human macrophages with *Mycobacterium tuberculosis* infection of Beijing/W and non-Beijing/W strain types. PloS One. 2015; 10(6): e0126018 10.1371/journal.pone.0126018 26053546 PMC4460131

[2] Baer CE, Rubin EJ, Sassetti CM. New insights into TB physiology suggest untapped therapeutic opportunities. Immunol Rev. 2015; 264(1): 327–43. 10.1111/imr.12267 25703570 PMC4339208

[3] Sebina I, Biraro IA, Dockrell HM, Elliott AM, Cose S. Circulating B-lymphocytes as potential biomarkers of tuberculosis infection activity. PloS One. 2014; 9(9): e106796 10.1371/journal.pone.0106796 25192196 PMC4156407

[4] Pollock KM, Montamat-Sicotte DJ, Grass L, Cooke GS, Kapembwa MS, Kon OM, et al PD-1 Expression and cytokine secretion profiles of *Mycobacterium tuberculosis*-Specific CD4^+^ T-cell subsets; potential correlates of containment in HIV-TB co-Infection. PloS One. 2016; 11(1): e0146905 10.1371/journal.pone.0146905 26756579 PMC4710462

[5] Booty MG, Nunes-Alves C, Carpenter SM, Jayaraman P, Behar SM. Multiple inflammatory cytokines converge to regulate CD8^+^ T cell expansion and function during tuberculosis. J Immunol. 2016; 196(4): 1822–31. 10.4049/jimmunol.1502206 26755819 PMC4799850

[6] Parida SK, Poiret T, Zhenjiang L, Meng Q, Heyckendorf J, Lange C, et al T-cell therapy: options for infectious diseases. Clin Infect Dis. 2015; 61 Suppl 3: S217–24. 10.1093/cid/civ615 PMC4583575 26409284

[7] Rao M, Valentini D, Poiret T, Dodoo E, Parida S, Zumla A, et al B in TB: B cells as mediators of clinically relevant immune responses in tuberculosis. Clin Infect Dis. 2015; 61 Suppl 3:S225–34. 10.1093/cid/civ614 PMC4583574 26409285

[8] Torrado E, Fountain JJ, Robinson RT, Martino CA, Pearl JE, Rangel-Moreno J, et al Differential and site specific impact of B cells in the protective immune response to *Mycobacterium tuberculosis* in the mouse. PloS One. 2013; 8(4): e61681 10.1371/journal.pone.0061681 23613902 PMC3627912

[9] Zhu Q, Zhang M, Shi M, Liu Y, Zhao Q, Wang W, et al Human B cells have an active phagocytic capability and undergo immune activation upon phagocytosis of *Mycobacterium tuberculosis*. Immunobiology. 2016; 221(4): 558–67. 10.1016/j.imbio.2015.12.003 26719096

[10] Achkar JM, Chan J, Casadevall A. Role of B cells and antibodies in acquired immunity against *Mycobacterium tuberculosis*. Cold Spring Harb Perspect Med. 2014; 5(3): a018432 10.1101/cshperspect.a018432 25301934 PMC4355258

[11] Kazemzadeh M, Safaralizadeh R, Orang AV. LncRNAs: emerging players in gene regulation and disease pathogenesis. J Genet. 2015; 94(4): 771–84. 26690535 10.1007/s12041-015-0561-6

[12] Uchida S, Dimmeler S. Long noncoding RNAs in cardiovascular diseases. Circ Res. 2015; 116(4): 737–50. 10.1161/CIRCRESAHA.116.302521 25677520

[13] Imam H, Bano AS, Patel P, Holla P, Jameel S. The lncRNA NRON modulates HIV-1 replication in a NFAT-dependent manner and is differentially regulated by early and late viral proteins. Sci Rep. 2015; 5: 8639 10.1038/srep08639 25728138 PMC4345339

[14] Yi Z, Li J, Gao K, Fu Y. Identifcation of differentially expressed long non-coding RNAs in CD4^+^ T cells response to latent tuberculosis infection. J Infect. 2014; 69(6): 558–68. 10.1016/j.jinf.2014.06.016 24975173 PMC7112653

[15] Pawar K, Hanisch C, Palma Vera SE, Einspanier R, Sharbati S. Down regulated lncRNA MEG3 eliminates mycobacteria in macrophages via autophagy. Sci Rep. 2016; 6: 19416 10.1038/srep19416 26757825 PMC4725832

[16] Wang Y, Zhong H, Xie X, Chen CY, Huang D, Shen L, et al Long noncoding RNA derived from CD244 signaling epigenetically controls CD8^+^ T-cell immune responses in tuberculosis infection. Proc Natl Acad Sci U S A. 2015; 112(29): E3883–92. 10.1073/pnas.1501662112 26150504 PMC4517270

[17] Jiang TT, Wei LL, Shi LY, Chen ZL, Wang C, Liu CM, et al Microarray expression profile analysis of mRNAs and long non-coding RNAs in pulmonary tuberculosis with different traditional Chinese medicine syndromes. BMC Complement Altern Med. 2016; 16(1): 472 10.1186/s12906-016-1436-y 27855662 PMC5114807

[18] Yang X, Yang J, Wang J, Wen Q, Wang H, He J, et al Microarray analysis of long noncoding RNA and mRNA expression profiles in human macrophages infected with *Mycobacterium tuberculosis*. Sci Rep. 2016; 6: 38963 10.1038/srep38963 27966580 PMC5155227

[19] Alam-Faruque Y, Huntley RP, Khodiyar VK, Camon EB, Dimmer EC, Sawford T, et al The impact of focused Gene Ontology curation of specific mammalian systems. PloS One. 2011; 6(12): e27541 10.1371/journal.pone.0027541 22174742 PMC3235096

[20] Goovaerts O, Jennes W, Massinga-Loembé M, Ondoa P, Ceulemans A, Vereecken C, et al Lower pre-treatment T cell activation in early- and late-onset tuberculosis-associated immune reconstitution inflammatory syndrome. PloS One. 2015; 10(7): e0133924 10.1371/journal.pone.0133924 26208109 PMC4514632

[21] Hu P, Yang J, Hou Y, Zhang H, Zeng Z, Zhao L, et al LncRNA expression signatures of twist-induced epithelial-to-mesenchymal transition in MCF10A cells. Cell Signal. 2014; 26(1): 83–93. 10.1016/j.cellsig.2013.10.001 24113349

[22] Yao Y, Li J, Wang L. Large intervening non-coding RNA HOTAIR is an indicator of poor prognosis and a therapeutic target in human cancers. Int J Mol Sci. 2014;15(10):18985–99. 10.3390/ijms151018985 25334066 PMC4227256

[23] Huang X, Hao C, Bao H, Wang M, Dai H. Aberrant expression of long noncoding RNAs in cumulus cells isolated from PCOS patients. Assist Reprod Genet. 2016; 33(1):111–21. 10.1007/s10815-015-0630-z PMC4717141 26650608

[24] Dang Y, Lan F, Ouyang X, Wang K, Lin Y, Yu Y, et al Expression and clinical significance of long non-coding RNA HNF1A-AS1 in human gastric cancer. World J Surg Oncol. 2015;13: 302 10.1186/s12957-015-0706-3 26472090 PMC4608159

[25] Feng J, Bi C, Clark BS, Mady R, Shah P, Kohtz JD. The Evf-2 noncoding RNA is transcribed from the Dlx-5/6 ultraconserved region and functions as a Dlx-2 transcriptional coactivator. Genes Dev. 2006; 20:1470–84. 10.1101/gad.1416106 16705037 PMC1475760

[26] He Y, Vogelstein B, Velculescu VE, Papadopoulos N, Kinzler KW. The antisense transcriptomes of human cells. Science. 2008; 322: 1855–7. 10.1126/science.1163853 19056939 PMC2824178

[27] Lin XC, Zhu Y, Chen WB, Lin LW, Chen DH, Huang JR, et al Integrated analysis of long nod-coding RNAs and mRNA expression profiles reveals the potential role of lncRNAsin gastric cancer pathogenesis. Int J Oncol. 2014; 45(2): 619–28. 10.3892/ijo.2014.2431 24819045

[28] Ørom UA, Derrien T, Beringer M, Gumireddy K, Gardini A, Bussotti G, et al Long noncoding RNAs with enhancer-like function in human cells. Cell. 2010; 143(1): 46–58. 10.1016/j.cell.2010.09.001 20887892 PMC4108080

[29] Wang N, Yao M, Xu J, Quan Y, Zhang K, Yang R, et al Autocrine activation of CHRM3 promotes prostate cancer growth and castration resistance via CaM/CaMKK- mediated phosphorylation of Akt. Clin Cancer Res. 2015; 21(20): 4676–85. 10.1158/1078-0432.CCR-14-3163 26071486

[30] Weymouth KS, Blanton SH, Powell T, Patel CV, Savill SA, Hecht JT. Functional assessment of clubfoot associated HOXA9, TPM1,and TPM2 variants suggests a potential gene regulation mechanism. Clin Orthop Relat Res. 2016; 474(7): 1726–35. 10.1007/s11999-016-4788-1 27020427 PMC4887369

[31] Ueta M, Tamiya G, Tokunaga K, Sotozono C, Ueki M, Sawai H, et al Epistatic interaction between Toll-like receptor 3 (TLR3) and prostaglandin E receptor 3 (PTGER3) genes. J Allergy Clin Immunol. 2012; 129(5):1413–6. 10.1016/j.jaci.2012.01.069 22421267

[32] He Y, Ou Z, Chen X, Zu X, Liu L, Li Y, et al LPS/TLR4 signaling enhances TGF-β response through downregulating BAMBI during prostatic hyperplasia. Sci Rep. 2016; 6: 27051 10.1038/srep27051 27243216 PMC4886686

[33] Rottenberg ME, Carow B. SOCS3 and STAT3, major controllers of the outcome of infection with *Mycobacterium tuberculosis*. Semin Immunol. 2014; 26(6): 518–32. 10.1016/j.smim.2014.10.004 25458989

[34] Kleinsteuber K, Heesch K, Schattling S, Sander-Juelch C, Mock U, Riecken K, et al SOCS3 promotes interleukin-17 expression of human T cells. Blood. 2012; 120(22): 4374–82. 10.1182/blood-2011-11-392738 23033269

[35] Narayana Y, Balaji KN. NOTCH1 up-regulation and signaling involved in *Mycobacterium bovis* BCG-induced SOCS3 expression in macrophages. J Biol Chem. 2008; 283 (18): 12501–11. 10.1074/jbc.M709960200 18332140

[36] Ashenafi S, Aderaye G, Bekele A, Zewdie M, Aseffa G, Hoang AT, et al Progression of clinical tuberculosis is associated with a Th2 immune response signature in combination with elevated levels of SOCS3. Clin Immunol. 2014; 151(2): 84–99. 10.1016/j.clim.2014.01.010 24584041

