# Supplementary figures and images for: Deregulated lncRNAs in B Cells from Patients with Active Tuberculosis
Source: PLoS One. 2017 Jan 26;12(1):e0170712. doi: 10.1371/journal.pone.0170712 (PMC5268381; doi:10.1371/journal.pone.0170712)

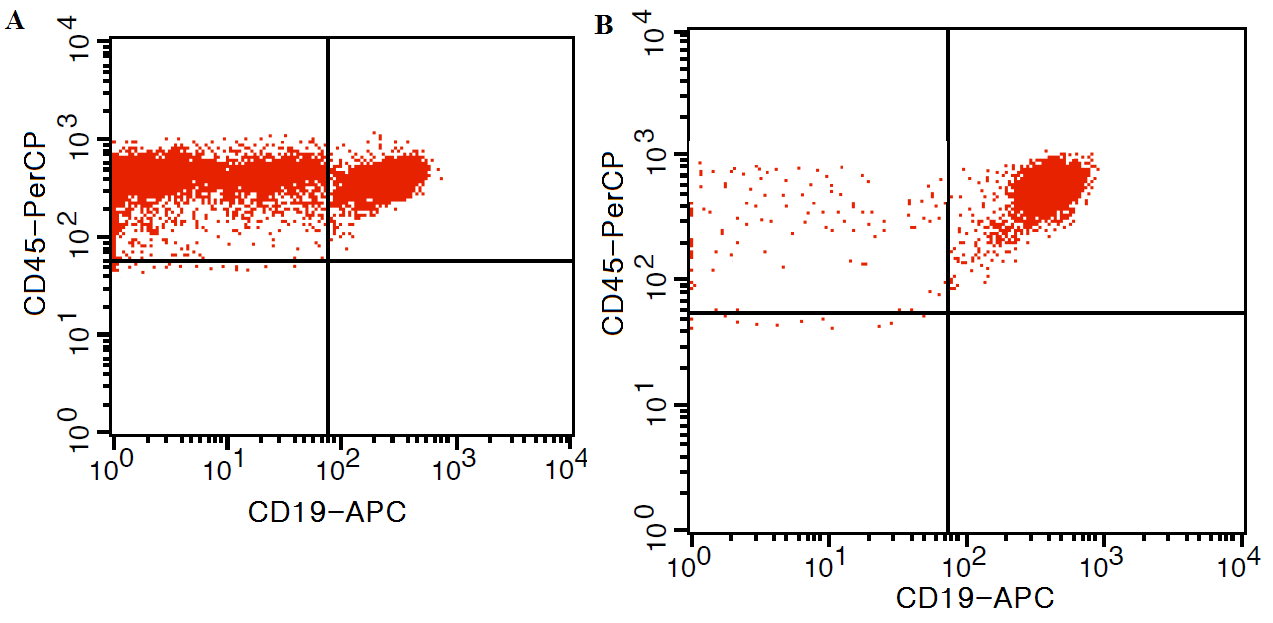

Supplement: S1 Fig — PBMCs before (A) and after (B) isolation of B cells. (TIF) [file pone.0170712.s001.TIF]
